# Effects of Supervised Exercise on the Development of Hypertensive Disorders of Pregnancy: A Systematic Review and Meta-Analysis

**DOI:** 10.3390/jcm11030793

**Published:** 2022-02-01

**Authors:** Marianna Danielli, Clare Gillies, Roisin Clare Thomas, Sarah Emily Melford, Philip Newton Baker, Thomas Yates, Kamlesh Khunti, Bee Kang Tan

**Affiliations:** 1 Department of Cardiovascular Sciences, University of Leicester, Leicester LE1 7RH, UK; md446@leicester.ac.uk (M.D.); rct21@leicester.ac.uk (R.C.T.); sm1056@leicester.ac.uk (S.E.M.); philip.baker@leicester.ac.uk (P.N.B.); 2 Diabetes Research Centre, Leicester General Hospital, Leicester LE5 4PW, UK; clg13@leicester.ac.uk (C.G.); ty20@leicester.ac.uk (T.Y.); kk22@leicester.ac.uk (K.K.); 3 National Institute for Health Research (NIHR) Leicester Biomedical Research Centre (BRC), Leicester General Hospital, Leicester LE5 4PW, UK; 4 NIHR Applied Research Collaboration—East Midlands (ARC-EM), Leicester General Hospital, Leicester LE5 4PW, UK

**Keywords:** exercise, gestational hypertension, physical activity, pre-eclampsia, systematic review, meta-analysis

## Abstract

Hypertensive disorders of pregnancy (HDP) are the most common medical complication in pregnancy, affecting approximately 10–15% of pregnancies worldwide. HDP are a major cause of maternal and perinatal morbidity and mortality, and each year, worldwide, around 70,000 mothers and 500,000 babies die because of HDP. Up-to-date high-quality systematic reviews quantifying the role of exercise and the risks of developing HDP are currently lacking. Physical exercise is considered to be safe and beneficial to pregnant women. Supervised exercise has been shown to be safe and to be more beneficial than unsupervised exercise in the general population, as well as during pregnancy in women with obesity and diabetes. Therefore, we undertook a systematic review and meta-analysis to investigate the effects of women performing supervised exercise during pregnancy compared to a control group (standard antenatal care or unsupervised exercise) on the development of HDP. We searched Medline, Embase, CINHAL, and the Cochrane Library, which were searched from inception to December 2021. We included only randomized controlled trials (RCTs) investigating the development of HDP compared to a control group (standard antenatal care or unsupervised exercise) in pregnant women performing supervised exercise. Two independent reviewers selected eligible trials for meta-analysis. Data collection and analyses were performed by two independent reviewers. The PROSPERO registration number is CRD42020176814. Of 6332 articles retrieved, 16 RCTs met the eligibility criteria, comparing a total of 5939 pregnant women (2904 pregnant women in the intervention group and 3035 controls). The risk for pregnant women to develop HDP was significantly reduced in the intervention compared to the control groups, with an estimated pooled cumulative incidence of developing HDP of 3% in the intervention groups (95% CI: 3 to 4) and of 5% in the control groups (95% CI: 5 to 6), and a pooled odds ratio (OR) comparing intervention to control of 0.54 (95% CI:0.40 to 0.72, *p* < 0.001). A combination of aerobic and anaerobic exercise, or yoga alone, had a greater beneficial effect compared to performing aerobic exercise only (mixed-OR = 0.50, 95% CI:0.33 to 0.75, *p* = 0.001; yoga-OR = 0.28, 95% CI:0.13 to 0.58, *p* = 0.001); aerobic exercise only-OR = 0.87, 95% CI:0.55 to 1.37, *p* = 0.539). Pregnancy is an opportunity for healthcare providers to promote positive health activities, thus optimizing the health of pregnant women with potential short- and long-term benefits for both mother and child. This systematic review and meta-analysis support a beneficial effect of either structured exercise (combination of aerobic, strength, and flexibility workouts) or yoga for preventing the onset of HDP. Yoga, considered a low-impact physical activity, could be more acceptable and safer for women in pregnancy in reducing the risk of developing HDP.

## 1. Introduction

Hypertension (high blood pressure) is the most common medical complication in pregnancy, affecting approximately 4–25% of pregnancies worldwide [1,2,3]. Hypertensive disorders of pregnancy (HDP) are a major cause of maternal and perinatal morbidity and mortality, and each year, worldwide, around 70,000 mothers and 500,000 babies die because of HDP [4,5]. Hypertension during pregnancy can be subdivided into chronic hypertension, gestational hypertension (GH), and the spectrum of pre-eclampsia (PE) [6]. Hypertension is defined as a systolic blood pressure equal or greater than 140 mmHg or diastolic blood pressure equal or greater than 90 mmHg [6]. If the systolic and diastolic blood pressure are equal or greater than 160 and 110, respectively, the hypertension is considered severe [6]. Chronic hypertension is hypertension found prior to pregnancy or before 20 weeks of gestation [6]. On the other hand, when hypertension is identified for the first time after 20 weeks of gestation and no proteins can be detected in the urine, a diagnosis of GH can be made [6]. Finally, PE is the new onset of hypertension after 20 weeks of gestation and proteinuria (≥30 mg/mmol in the urine collection or albumin/creatinine ≥8 mg/mmol or dipstick reading ≥1) [6]. HELLP (hemolysis, elevated liver enzymes, and low platelets) syndrome and eclampsia are severe forms of PE [6].

Women who develop GH are at increased risk of developing PE; approximately 60% who had GH before 28 weeks’ gestation developed PE, 26% between 28–33 weeks, 27% between 34–36 weeks, and 12% after 37 weeks’ gestation [7]. PE has significantly higher perinatal morbidity and mortality compared to GH [1,2,3]. The incidence of PE amongst pregnant women is roughly 5–8% [1,2,3]. Furthermore, the life expectancy of women who develop pre-eclampsia is reduced on average by 10 years [8], due to cardiovascular and cerebrovascular conditions later in life [9]. Finally, children born to women with PE have increased risks of developing metabolic and cardiovascular diseases later in life [10]. Thus, preventing the development of HDP could prevent the risks of long-term health issues for both mother and child.

Physical exercise is considered to be safe and beneficial to pregnant women [11]. However, there remains a paucity of research examining the effects of exercise in mitigating pregnancy complications. Supervised exercise has been shown to be safe and to be more beneficial than unsupervised exercise in the general population, as well as for women during pregnancy [12,13,14,15,16,17,18].

Up-to-date high quality systematic reviews quantifying the role of supervised exercise and the risk of developing HDP are currently lacking, and no previous reviews have conducted sub-group analyses by type of exercise intervention or by the setting in which interventions were delivered.

Therefore, we undertook a systematic review and meta-analysis to investigate the effects of women performing supervised exercise during pregnancy compared to a control group (standard antenatal care or unsupervised exercise) on the development of HDP.

## 2. Material and Methods

### 2.1. Search Strategy and Selection Criteria

This systematic review is reported following the Preferred Reporting Items for Systematic Reviews and Meta-Analyses (PRISMA) [19]. The PROSPERO registration number is CRD42020176814. The following databases were searched: Ovid MEDLINE, EMBASE, CINHAL, and the Cochrane Library from inception to November 2021. The search strategy included terms related to “exercise” and “physical activity” combined with terms related to “HDP”. The search was not limited by language. The complete strategy can be found in the Supplementary Material (Supplementary Figure S1).

Two reviewers (M.D., R.T.) independently screened the title and abstracts of all papers and, according to their relevance, obtained full text reports. Randomized controlled trials (RCTs) comparing any type of supervised exercise training in pregnancy with a control group of pregnant women receiving standard antenatal care in which the onset of HDP was a reported outcome have been included. Supervised exercise was defined as structured training planned and performed under professional supervision. Inclusion criteria have been set up as: (1) studies reporting original data (conference abstracts, case reports, case series, letters, editorials, guidelines, theses, commentaries, reviews, systematic reviews have been excluded); (2) RCTs; (3) studies performed on human participants; (4) studies performed on pregnant women; (5) studies with a control group; (6) studies in which exercise was performed with supervision and adherence was not self-reported by patients (via questionnaires, surveys, or telephone interviews). Both RefWorks and EndNote were used to manage the search results. Any disagreement during the process was resolved through discussion and, when necessary, by the advice of a third reviewer (C.G.).

Data was extracted independently by two reviewers following the Cochrane Handbook guidelines [20], and findings were reported according to PRISMA guidelines [19]. Consensus among all authors resolved any disagreement regarding papers to be included in the analysis.

As shown in Supplementary Table S1, exercise volume was assessed for each study by extracting data regarding exercise frequency (defined as the number of exercise sessions per week), weekly duration of the exercise program (expressed in minutes), total number of weeks of exercise spent in the program, and cumulative number of sessions. Data on adherence and exercise intensity were also extracted.

### 2.2. Data Analysis

For included studies, the cumulative incidence (%) for the intervention and control groups were calculated separately, and odds ratios comparing groups were pooled using random effects meta-analysis models to allow for between-study heterogeneity. Where studies reported an adjusted odds ratio, this was used in the analysis; if no odds ratio was reported, it was calculated using reported raw numbers. If no events occurred in any of the study arms, a continuity correction of 0.5 was applied [20]. Between-study heterogeneity was assessed using Higgins *I*^2^ statistic [21] and explored using meta-regression analysis to assess the effect of mean age, mean BMI, percentage of participants who were nulliparous, minutes per week of exercise recommended for the intervention, and publication year, on the estimated effect size.

Sub-group analyses were also carried out by type of physical exercise performed, type of HDP assessed, main setting in which the study was performed, and geographic area (continent). All analyses were carried out using Stata/IC 16·0. The risk of publication bias was assessed using funnel plots and Begg’s and Egger’s tests, as shown in the Supplementary Figure S2.

The quality of the included studies was assessed using the Cochrane Risk of Bias Tool for RCTs [22,23], as illustrated in Supplementary Tables S2 and S3. A sensitivity analysis was carried out including studies found to be at low risk of bias for all domains.

## 3. Results

**Figure 1:**
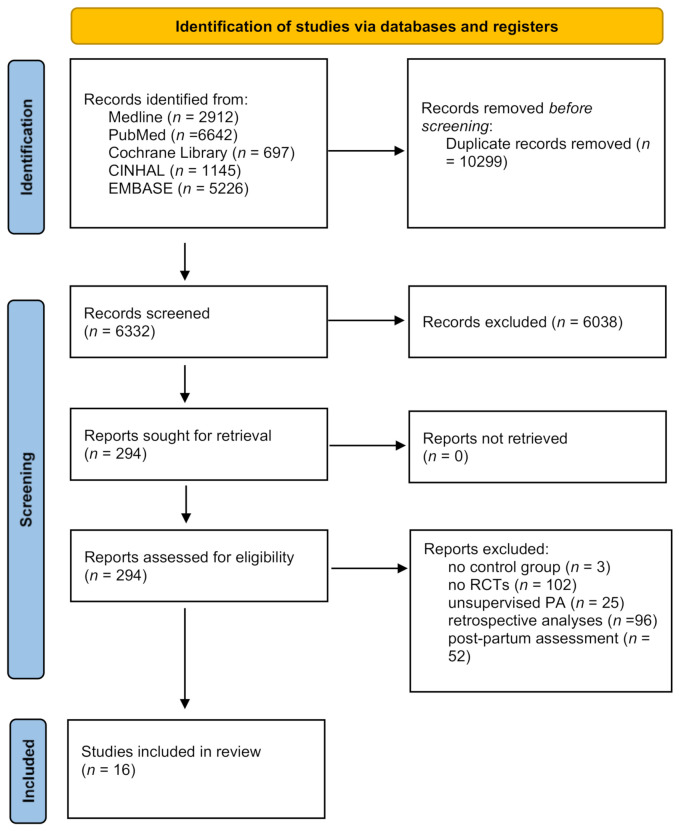
PRISMA (Preferred Reporting Items for Systematic Reviews and Meta-Analyses) flow chart of literature search.

A PRISMA flow chart of the literature search is shown in Figure 1. The search strategy returned 16,631 records in total. Duplicate papers were removed manually after sorting all papers by title. Of the 6332 evaluated papers, 6038 papers were excluded by title and abstract as the papers did not meet the selection criteria; of these, the full text of 368 of them was written in a foreign language (Bulgarian, Chinese, Croatian, Czech, Danish, Dutch, French, German, Hebrew, Hungarian, Italian, Japanese, Norwegian, Polish, Portuguese, Russian, Serbian, Spanish, Swedish, Ukrainian); these papers have been screened by title and abstract only (in English) and then excluded from the full text screening because they did not fulfil the inclusion criteria of our systematic review. Two hundred and ninety-four papers were selected by full text; of these, 278 were excluded; of these, 57 were studies in which exercise was performed with no direct supervision, and, finally, 3 did not have a control group [24,25,26]. Cross-checking was performed for all articles by a second reviewer. Overall, 16 RCTs met the inclusion criteria for this systematic review [27,28,29,30,31,32,33,34,35,36,37,38,39,40,41,42]. The majority of included studies were based in a hospital setting (*n* = 11), 4 in universities, and 1 in multiple settings (hospital, university, and primary care units). Studies were based in Europe (Spain *n* = 4, Croatia *n* = 1, Norway *n* = 2), America (USA *n* = 2, Brazil *n* = 3), and Asia (India *n* = 3, China = 1). Across studies, the mean age varied from 24.7 to 32.3 years, and mean BMI pre-pregnancy from 22.9 to 33.1 kg/m^2^. Except for one conducted in the year 2000 [38], all studies were carried out between 2012 and 2020. The studies’ sizes ranged from 16 to 1348. Further details of study characteristics can be found in Supplementary Table S4.

**Figure 2:**
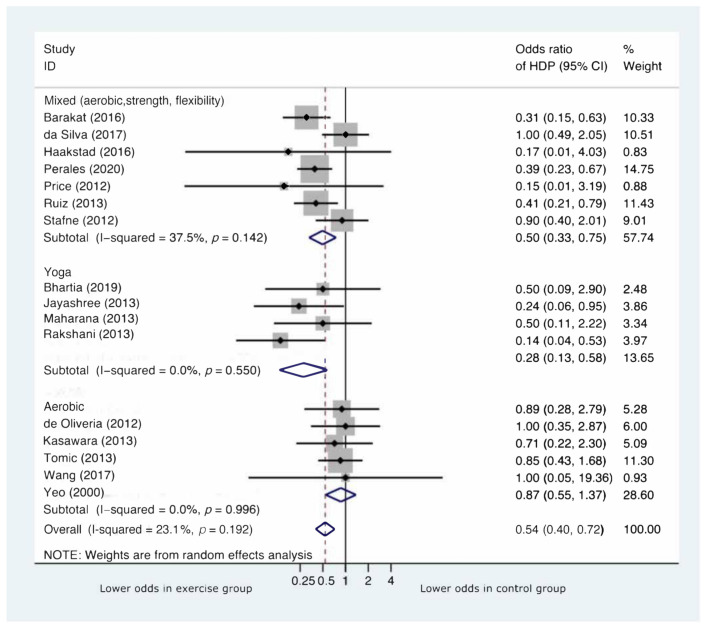
Forest plot by type of physical exercise performed.

As shown in Supplementary Table S1, four out of sixteen studies did not report data on participants’ adherence. When reported, adherence was <75% in two RCTs (da Silva et al., 2017 (56.25%); Stafne et al., 2012 (55%)). In both studies, the sample size was high (639 and 855, respectively), and the type of intervention was a mixture of aerobic and anaerobic activity [30,40]. The last factor is probably the one that accounts the most for the lowest study compliance since the type of physical exercise was more complex than performing yoga or aerobic exercise only. Furthermore, the vast majority of RCTs in the literature on physical exercise during pregnancy used unsupervised exercise as the intervention, meaning that no real data on adherence can be provided. Interestingly, the ORs (95%) of developing HDP in the study groups were 1.00 (0.49, 2.05) and 0.90 (0.40, 2.01), respectively (as shown in Figure 2). This finding suggests an important role for participants’ adherence in exercise effectiveness.

Exercise intensity has been reported in all studies with aerobic activity as intervention (see Supplementary Table S1) and has been measured using the Borg Rate of Perceived Exertion (RPE) has been used as measure of exercise intensity (https://academic.oup.com/occmed/article/67/5/404/3975235) (link last accessed on 15 December 2021). The scale ranges from 6 (‘no exertion at all’) to 20 (‘maximum exertion’) (https://www.cdc.gov./physicalactivity/basics/measuring/exertion.htm?CDC_AA_refVal=https%3A%2F%2Fwww.cdc.gov%2Fphysicalactivity%2Feveryone%2Fmeasuring%2Fexertion.html) (link last accessed on 15 December 2021). In nine out of the twelve included studies reporting data on exercise intensity, the level of exertion has been reported as moderate, with RPE levels ranging from 11.9 to 16. Pregnant women performed light-to-moderate exercise (RPE = 10–12) in two RCTs (Ruiz et al., 2013 and Perales et al., 2020) [34,39], and low-intensity activity in one study (Kasawara et al., 2013) [42]. Heart rate has been considered an additional indicator of exercise intensity throughout the exercise programme in eight studies, and in Kasawara et al., 2013, this was the only parameter of exertion levels [42]. Regarding the studies involving yoga, we were unable to report RPE levels, as yoga is a combination of breathing and loosening exercises, deep relaxation, meditation, asana postures, and pranayama. The aerobic exercise components of yoga could translate to light-to-moderate exercise and may be the main factor for lowering heart rate and blood pressure. However, our findings show that yoga is more effective in reducing the risk of developing HDP than the other structured exercises, which suggests that breathing exercises and/or meditation (and anaerobic exercise too) could play a pivotal role in reducing the risk of HDP.

No major adverse event related to performing exercise was reported in any of the analyzed RCTs. HDP assessment was mainly physician-assessed (7 studies), self-reported (2 studies), taken from medical record (3 studies), or not specified (4 studies). The main outcomes of interest were PE and GH. Notably, eclampsia was reported in two studies [35,36]; in both studies, it affected women only in the control group.

Six thousand and fifty-four pregnant women (2976 pregnant women in the intervention group and 3078 controls) were included in the 16 studies. The cumulative incidence of developing HDP (Supplementary Table S4) was 3% in the intervention groups (95% CI:3 to 4) and 5% in the control groups (95% CI:5 to 6). Furthermore, supervised exercise performed during pregnancy significantly reduced the risk of developing HDP (OR = 0.54, 95% CI:0.40 to 0.72, *p* < 0.001). Of the 16 studies included in the meta-analysis, 13 estimated an effect size that favored the intervention, with 5 studies showing a significant effect (Figure 2).

Analyses by type of physical exercise performed by the participants showed that many modes of exercise were used (walking on a treadmill, cycling on a stationary bicycle, using elliptical trainers, or circuit training). All four studies examining yoga as an intervention included breathing exercises, yogic postures, and meditative exercise with deep relaxation techniques (Supplementary Table S4). As shown in Figure 2, participants performing either mixed exercise of both aerobic and anaerobic exercise (OR = 0.50, 95% CI:0.33 to 0.75, *p* = 0.001) or yoga alone (OR = 0.28, 95% CI:0.13 to 0.58, *p* = 0.001) had the most significant risk reduction of HDP compared to those performing aerobic exercise only (OR = 0.87, 95% CI:0.55 to 1.37, *p* = 0.539). A summary of the comparison between subgroups is shown in Table 1.

**Figure 3:**
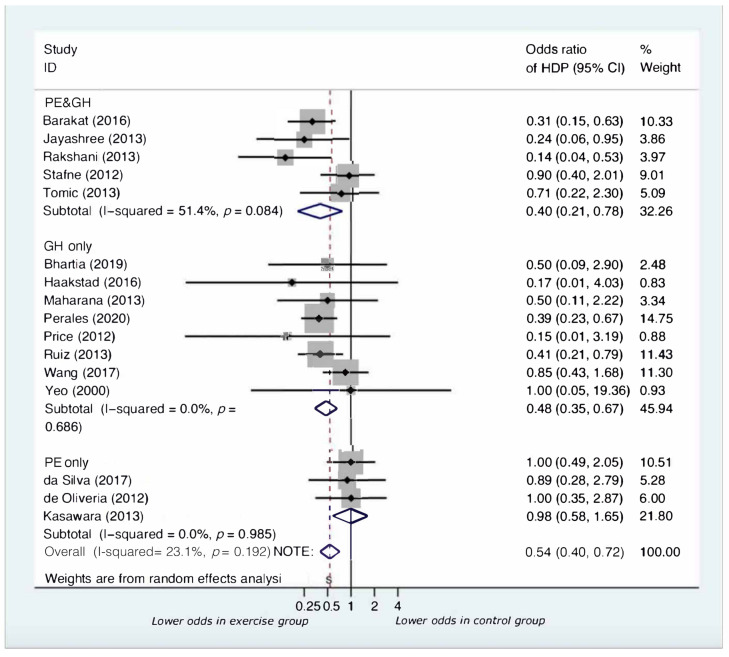
Forest plot for subgroup analysis by type of HDP.

Analyses by type of HDP (Figure 3) showed that supervised exercise interventions significantly reduced the risk of developing GH (OR = 0.48, 95% CI:0.35 to 0.67, *p* < 0.001) and for PE and GH (OR = 0.40, 95% CI:0.21 to 0.78, *p* = 0.007). The impact of exercise on the development of PE was not significant (OR = 0.98, 95% CI: 0.58 to 1.65, *p* = 0.928). No significant differences were found between the sub-group pooled odds ratios (Table 1).

**Figure 4:**
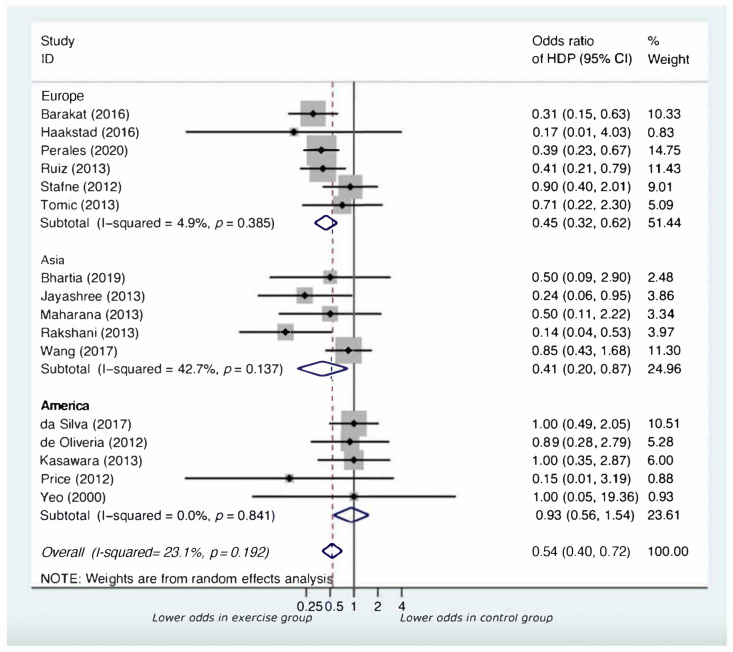
Forest plot for subgroup analysis by continent.

Analyses by continent demonstrated that the impact of supervised exercise on the development of HDP was similar across continents (Europe-OR = 0.45, 95% CI:0.32 to 0.62, *p* < 0.001; Asia-OR = 0.41, 95% CI:0.20 to 0.87, *p* = 0.020; America-OR = 0.93, 95% CI:0.56 to 1.54, *p* = 0.770), as shown in Figure 4 and Table 1.

**Figure 5:**
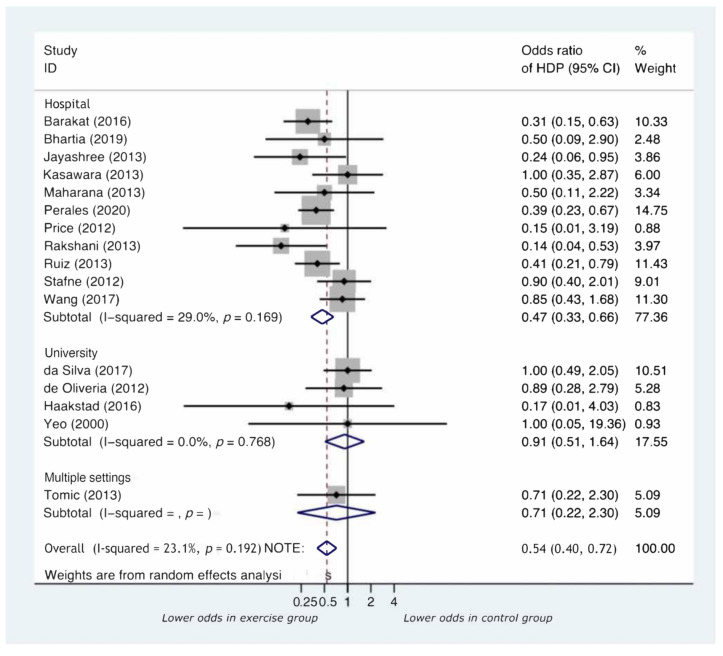
Forest plot for subgroup analysis by setting.

Analyses by setting (Figure 5) showed that only supervised interventions undertaken in a hospital setting showed a significant reduction in the risk of developing HDP (hospital-OR = 0.47, 95% CI:0.33 to 0.66, *p* < 0.001). Interventions conducted in university (OR = 0.91, 95% CI:0.51 to 1.64, *p* = 0.761) or multiple settings-OR = 0.71, 95% CI:0.22 to 2.30, *p* = 0.572) were not significant.

Meta-regression analyses did not find any statistically relevant association between study effect size and mean age, mean BMI, publication year, nulliparity, and recommended minutes of supervised exercise performed per week (Table 2).

According to the funnel plot and Egger’s and Begg’s tests (*p* = 0.618 and *p* = 0.392 respectively), no statistically significant publication bias was present, as illustrated in Supplementary Figure S1. The majority of studies were scored as being at low risk of bias for the domains of random sequence generation, incomplete data outcome, outcome measurement, and selective reporting. Full details of the quality assessment are presented in Supplementary Table S3. The results of the sensitivity analyses by study quality assessment found no substantial change in the results when only those studies at low risk of bias have been included (pooled odds ratio 0.51 (0.34, 0.79), *p* = 0.002).

## 4. Discussion

Pregnancy is an opportunity for healthcare providers to promote positive health activities, thus optimizing the health of pregnant women with potential short- and long-term benefits for both mother and child. Physical exercise is considered to be safe and beneficial to pregnant women; the Royal College of Obstetricians and Gynaecologists (RCOG) general advice is that healthy pregnant women should perform at least 150 min per week of moderate intensity activity, including leisure-time physical activity, as well as outdoor and indoor exercise [11]. In addition, the official guidelines of several other countries also encourage women to perform physical exercise during pregnancy [43]. Similarly, the antenatal care for uncomplicated pregnancy NICE guidelines state that “pregnant women should be informed that beginning or continuing a moderate course of exercise during pregnancy is not associated with adverse outcomes” [44].

Research has been conducted assessing the effects of physical exercise on pregnancy [45,46,47], but few have focused on the effects of exercise on the development of HDP [48,49]. The results of these studies have been mixed. In most cases, exercise has been reported to be beneficial [24,50,51,52], but in some, the results have been inconclusive [53]. Furthermore, the vast majority of studies only focused on one particular type of physical exertion at a time [50,52]. An umbrella review reported only weak evidence, suggesting an inverse correlation between physical activity and the odds of developing hypertension or depression during pregnancy [54]. Similarly, a meta-analysis of 15 studies by Aune et al. demonstrated that exercise reduced the risk of developing PE [55]. Moreover, a meta-analysis showed that exercise lowered the risk of developing gestational diabetes mellitus, GH, and PE [56]. Conversely, Syngelaki et al. reported that, although diet and exercise were beneficial for weight control during pregnancy, they did not seem to reduce the risk of developing PE [57]. Wolf et al. performed a systematic review of the literature, and, among the 11 studies analyzed, only 1 study demonstrated a detrimental effect of leisure-time exercise on the risk of developing PE [58]. Physical exercise during pregnancy could prevent excessive gestational weight gain, promote insulin sensitivity, and reduce systemic inflammation and oxidative stress, leading to improved endothelial function, as well as promoting placental angiogenesis, factors that lower the risks of developing PE during pregnancy [49].

This systematic review and meta-analysis of 16 RCTs in a variety of settings showed that a structured exercise program or yoga significantly reduces the risk of HDP. Interestingly, the extent of this reduction is similar between pregnant women performing yoga or a combination of aerobic and anaerobic exercise. All four studies examining yoga as an intervention included breathing exercises, yogic postures, and meditative exercise with deep relaxation techniques. Furthermore, analyses by setting showed that only supervised interventions undertaken in a hospital setting showed a significant reduction in the risk of developing HDP. Our analyses also demonstrate that the effect of supervised exercise interventions did not differ by continent or type of HDP considered. Finally, meta-regression analyses showed that the impact of supervised exercise interventions was not associated with study level characteristics, including mean age, mean BMI, publication year, nulliparity, or minutes of recommended exercise per week.

Mixed (combination of aerobic and anaerobic exercise) and yoga were found to more beneficial than aerobic exercise alone in lowering the risk of HDP. Anaerobic exercise promotes muscle mass and strength, and this in turn has been reported to protect against hypertension [59]. In addition, breathing exercises in yoga are associated with a reduction in vagal tone, leading to a lower heart rate and blood pressure [60]. Furthermore, high cortisol levels are associated with high blood pressure [61]; yoga has been shown to reduce cortisol and consequently blood pressure [62]. These are plausible mechanisms that may explain our findings. Finally, we find our observations on interventions undertaken in a hospital interesting but unable to afford a reason to explain this finding.

To the best of our knowledge, this is the first systematic review and meta-analysis comparing the beneficial effects of different types of supervised exercise on the development of HDP including data derived only from RCTs. Additionally, we included only studies in which exercise was performed under supervision, with the aim of including only high-quality evidence that could be reproducible by other researchers. Supervised exercise has been shown to be safe and to be more beneficial than unsupervised exercise in the general population and for women during pregnancy [12,13,14]. Study conducted on small groups of pregnant women demonstrated how supervised aerobic exercise improves glucose tolerance, physical fitness, and glycemic control [15,16,17]. Similarly, supervised exercise seemed to be beneficial and safe for obese and overweight pregnant women [18]. Previous reviews did not restrict their criteria to supervised exercise only [55,57,58]. Also, in contrast to previous reviews, we included only RCTs assessing the effects of supervised exercise on pregnancies at high risk of developing HDP [55,57,58]. Finally, the beneficial effects of exercise could also apply to cases of essential hypertension diagnosed before pregnancy.

The strengths of this systematic review and meta-analysis are that robust methods have been utilized throughout, including a comprehensive search of multiple databases; publication bias and study quality have both been assessed, and meta-analysis methodologies have been used to pool results across studies. However, this systematic review did not use individual patient data, thus it would be difficult to appreciate clinical differences between single patients. Furthermore, the number of studies assessed was moderate, and this was probably due to the selective inclusion criteria used needed to ensure the specificity of our research question and to derive the most meaningful high-quality data.

For future research, we suggest that larger RCTs be designed to compare specific interventions, commencing in different pregnancy trimesters, of both supervised and unsupervised exercise and their compliance (considering reasons that could affect compliance and retention of study participants) in multiple settings and in different populations, with longer follow-up time to delivery for best practice of the management of women with HDP to directly assess the effects of different supervised exercise interventions on the risk of developing HDP.

## 5. Conclusions

Pregnancy is an opportunistic time for healthcare providers to promote positive health activities, thus optimizing the health of pregnant women with potential short- and long-term benefits for both mother and child. To the best of our knowledge, this is the most up-to-date systematic review and meta-analysis with high-quality data (including only RCTs) demonstrating the benefits of supervised exercise in terms of reducing the risk of developing HDP, with yoga or structured training (comprising of aerobic, strength, and flexibility workouts) being more beneficial than performing aerobic activities alone. Yoga, considered a low-impact physical activity, could be more acceptable and safer for women in pregnancy in reducing the risk of developing HDP. This systematic review and meta-analysis pave the way for more specific recommendations and research into lifestyle interventions during pregnancy to mitigate both short- and long-term health sequelae in both mother and child.

## Figures and Tables

**Table 1:** Summary of subgroup analyses performed.

Categories	Subgroups	Studies (*n*)	OR (95% CI), *p*-Value	I^2^	Comparison between Sub-Groups (*p*-Value)
**All**		16	0.54 (0.40, 0.72), *p* < 0.001	23.1%	
**Type of exercise**	Yoga	4	0.28 (0.13, 0.58) *p* = 0.001	0.0%	Reference
	Aerobic	5	0.87 (0.55, 1.37) *p* = 0·539	23.1%	0.031
	Mixed	7	0.50 (0.33, 0.75) *p* = 0.001	37.5%	0.203
**Type of HDP**	PE only	3	0.98 (0.58, 1.65), *p* = 0.928	0.0%	Reference
	PE & GH	5	0.40 (0.21, 0.78), *p* = 0.007	51.4%	0.053
	GH only	8	0.48 (0.35, 0.67), *p* < 0.001	0.0%	0·083
**Region**	America	5	0.93 (0.56, 1.54), *p* = 0.770	0.0%	Reference
	Europe	6	0.45 (0.32, 0.62) *p* < 0.001	4.9%	0.885
	Asia	5	0.42 (0.20, 0.87), *p* = 0.020	42.7%	0.065
**Setting**	Hospital	11	0.47 (0.33, 0.66), *p* < 0.001	29.0%	Reference
	University	4	0.91 (0.51, 1.64), *p* = 0.761	0.0%	0.124
	Multiple settings	1	0.71 (0.22, 2.30) *p* = 0.572		0.546

**Table 2:** Results of meta-regression models.

Study Level Variable	Coefficient (95% CI)	*p*-Value
Mean age	0.98 (0.84, 1.14)	0.745
Mean BMI	1.04 (0.91, 1.19)	0.495
Publication year	0.98 (0.88, 1.09)	0.695
Percentage nulliparous	0.99 (0.96, 1.02)	0.634
Recommended minutes of exercise	0.99 (0.99, 1.00)	0.473

## Data Availability

All data underlying this article are available in the article and in its online supplementary material. We will willingly share our knowledge, protocol, and expertise when asked.

## Supplementary Materials

The following are available online at https://www.mdpi.com/article/10.3390/jcm11030793/s1, Supplementary Figure S1: Search Strategy (Medline); Supplementary Figure S2—Funnel plot for publication bias; Supplementary Table S1: Characteristics of the exercises in the included studies; Supplementary Table S2: Quality assessment using the Cochrane risk of bias tool for randomized controlled trials; Supplementary Table S3: Assessment of the risk of bias for each important outcome within studies; Supplementary Table S4: Characteristics of the included studies.
