# Supplementary material for: Effects of Supervised Exercise on the Development of Hypertensive Disorders of Pregnancy: A Systematic Review and Meta-Analysis
Source: J Clin Med. 2022 Feb 1;11(3):793. doi: 10.3390/jcm11030793 (PMC8836524; doi:10.3390/jcm11030793)
Supplement: Supplementary file 1 [file jcm-11-00793-s001.zip › jcm-1583915 suppl/Supplementary Figure S1.pdf]

Supplementary Figure S1. Search Strategy (Medline).

"physic* exercis*" OR exercis* OR "physic* activ*" OR physic* activ* OR physic* effort* OR exp "physical exertion"/ OR train* OR fit* OR fitness OR sport* OR streng* OR exp "cool-down exercise"/ OR exp gymnastic/ OR exp "muscle stretching exercise"/ OR exp running/ OR exp swimming/ OR exp walking/ OR exp "warm-up exercise"/ OR exp "resistance training"/ OR exp "plyometric exercise"/ OR exp "circuit-based exercise"/ OR exp exercise/ OR exp "exercise movement techniques"/ OR exp sports/ OR exp running/ OR exp jogging/ OR run* OR jog* OR walk* OR stretch* OR tennis OR athletic* OR bicycle OR cycle OR cycling OR tai chi OR yoga OR pilates OR climb* OR workout* OR danc* OR ballet* OR box* OR kickbox* OR "weight* lift*"

AND

pre-eclamp* OR preeclamp* OR tox?emia* OR exp "hypertension, pregnancy-induced"/ OR exp "pre-eclampsia"/ OR exp toxemia/ OR eclamp* OR exp eclampsia/ OR edema-proteinuria-hypertension gestosis OR edema proteinuria hypertension gestosis OR exp "HELLP syndrome"/ OR pregnancy induced hypertension OR Gestational Hypertension OR Transient Hypertension, Pregnancy OR EPH Complex OR EPH Gestosis OR Hypertension Edema Proteinuria Gestosis OR pregnan* AND hypertens*
