# Supplementary material for: Effects of Supervised Exercise on the Development of Hypertensive Disorders of Pregnancy: A Systematic Review and Meta-Analysis
Source: J Clin Med. 2022 Feb 1;11(3):793. doi: 10.3390/jcm11030793 (PMC8836524; doi:10.3390/jcm11030793)
Supplement: Supplementary file 1 [file jcm-11-00793-s001.zip › jcm-1583915 suppl/Supplementary Table S1.pdf]

Supplementary Table S1. Characteristics of the exercises in the included studies.

Study	Control Group Intervention	Exercise Group Intervention	Inclusion Criteria	Exclusion Criteria	Frequency (sessions/week)	Weekly duration (mins)	Programme duration (weeks)	Sessions (n)	Adherence	Aerobic exercise Intensity
**Barakat et al., 2016**	standard antenatal care + advice of positive effects of exercise	warm-up, resistance, strength, cool-down	low-risk pregnancy	history of risk of preterm delivery, GDM, T1DM, T2DM, multiple or complicated pregnancy	3	150-165	around 30	85	≥ 80%	moderate (RPE= 12-14, HR<70% of maximum HR)
**Bhartia et al., 2019**	standard antenatal care	yoga	low-risk pregnancy	high-risk pregnancy	3	150	12	36	not reported	n/a
**de Oliveira et al., 2012**	standard antenatal care	warm-up and walking	healthy pregnancy, pregnancy, sedentary on admission, GW< 13, singleton	smoking, chronic disease, history of premature delivery, fetal abnormalities, placenta praevia, history of vaginal bleeding, hematomas, PROM, cervical length < 2.5 cm	3	variable and gradually increased	12 or 19	36 or 57	≥ 85%	moderate (RPE=12-16, HR=60-80% of maximum HR)
**da Silva et al., 2017**	standard antenatal care	warm-up, aerobic, strength, stretching	healthy pregnancy, maternal age >18, not participating in exercise programmes	hypertension, CV disease, diabetes, history of miscarriage or preterm birth, IVF, multiple pregnancy, persistent bleeding, smoking (>20 cigarettes daily), BMI >35	3	180	>16	>48	56.25%	moderate (RPE=12-14)
**Haakstad et al., 2016**	standard antenatal care	warm-up, aerobic dance,	healthy pregnancy, formerly inactive	high-risk pregnancy	>2	>120	>12	>24	83.30%	moderate (RPE=12-14,

		strength, cool-down								HR<85% of maximum HR)
**Jayashree et al., 2013**	standard antenatal care + unsupervised walking	yoga	high-risk pregnancy (multiple pregnancy, maternal age <20 or >35, BMI>30, genetic history of pregnancy complications)	normal pregnancy, anemia, history of clotting disorders, renal/hepatic/heart disease, seizure disorders, structural abnormalities in the pelvis	3	not reported	12	36	not reported	n/a
**Kasawara et al., 2013**	standard antenatal care	aerobic exercise (stationary bicycle)	chronic hypertension and/or history of pre-eclampsia	multiple pregnancy, cervical insufficiency, vaginal bleeding, heart disease, SLE, kidney failure, neurological disorders, engaged in supervised PA	1	30	not reported	9.24	not reported	low (HR=20% above resting HR)
**Maharana et al., 2013**	standard antenatal care + advised to stretching	yoga	healthy pregnancy between 18 and 20 GW, live previous children, primi- or secundi-gravida, previous NVD	medical problems, multiple pregnancy, IVF, maternal physical abnormalities, fetal abnormalities, previous exposure to yoga or intense PA	variable	variable	not reported	not reported	95%	n/a

**Perales et al., 2020**	standard antenatal care + nutrition & PA counselling	low-impact dance, stretching, toning/resistance training, pelvic floor muscle training	healthy pregnancy, singleton, maternal age between 18 and 35	any contraindication to perform PA	3	150-165	29-30	87-90	>95%	light-to-moderate (RPE=10-12, HR <60% of maximum HR)
**Price et al., 2012**	standard antenatal care	step aerobics (day 1), walking (day 3), circuit training (day 3)	singleton, BMI<39,	aerobic exercise for more than once a week for the past 6 months, chronic heart/lung disease, poorly controlled diabetes, hypertension, epilepsy, hyperthyroidism, severe anemia, orthopedic limitations, history of premature delivery, SGA or miscarriage	4	180-240	variable	variable	77%	moderate (RPE=12-14)
**Rakhshani et al., 2013**	standard antenatal care + conventional antenatal exercise advice (walking)	yoga	<12 GW + risk factors (personal or familiar history of poor obstetrics outcome, multiple pregnancy, <20 or >35 yo, BMI>30)	severe renal, hepatic, gallbladder or heart disease, structural abnormalities of the respiratory system, hereditary anemia, seizures, STDs, contraindications to perform PA	3	180	15	45	not reported	n/a
**Ruiz et al., 2013**	standard antenatal care +	warm-up, aerobic (dance),	sedentary, single and uncomplicated	any contraindication to perform PA	3	150-165	29-30	85	>97%	light-to-moderate (RPE=10-12, HR

	nutrition & PA counselling	resistance, cool-down	pregnancy, no high risk of preterm delivery							<60% of maximum HR)
**Stafne et al., 2012**	standard antenatal care + nutrition & PA counselling	aerobic (low-impact dance), strength, stretching	white women, aged 18 or older, singleton live fetus	high-risk pregnancy and/or disease that could interfere with participation, living more than 30-minutes' drive from the hospitals	1	60	12	12	55%	moderate (RPE=13-14)
**Tomic et al., 2013**	standard antenatal care	warm-up, aerobic exercise, stretching, cool-down	normal pregnancy, good general health, maternal age between 18 and 35	chronic/acute medical conditions, multiple gestation, persistent bleeding (II or III trimester), placenta praevia (after 26 weeks), poorly controlled hypertension, diabetes, thyroid disease, incompetent cervix, recurrent miscarriage, heavy smokers	3	150	variable	variable	>80%	moderate (RPE=11.9, HR=60-75% of maximum HR)
**Wang et al., 2017**	standard antenatal care	warm-up, stationary cycling, cool-down	singleton, non-smoking, BMI>/=24, >18 yo, a2+6 weeks gestation	cervical insufficiency, ongoing medication for pre-existing hypertension, diabetes, cardiac disease, systemic lupus erythematosus, thyroid disease, psychosis, ongoing treatment with metformin or corticosteroids, contraindications to PA	3	90	26-27	78-81	>80% (in 90% of participants)	moderate (RPE=12-14, HR=65-75% of maximum HR)

**Yeo et al., 2000**	standard antenatal care	warm-up, steady state (bicycle ergometer or treadmill), cool-down	history of mild hypertension, GH or family history of hypertensive disorders, <14 GW, maternal age >18	diabetes, renal disease, multiple pregnancy, extremely vigorous exercisers	3	90	10	30	90%	moderate (RPE=13)

GDM=Gestational Diabetes Mellitus; T1DM=Type 1 Diabetes Mellitus; T2DM=Type 2 Diabetes Mellitus2; PROM=Premature Rupture of Membranes; IVF= In Vitro Fertilization; SLE=Systemic Lupus Erythematosus; PA=Physical Activity; NVD=Normal Vaginal Delivery; SGA=Small for Gestational Age; STDs=Sexual Transmitted Diseases; RPE=Rate of Perceived Exertion; HR=Heart Rate; n/a=not applicable.
