# Supplementary material for: Effects of Supervised Exercise on the Development of Hypertensive Disorders of Pregnancy: A Systematic Review and Meta-Analysis
Source: J Clin Med. 2022 Feb 1;11(3):793. doi: 10.3390/jcm11030793 (PMC8836524; doi:10.3390/jcm11030793)
Supplement: Supplementary file 1 [file jcm-11-00793-s001.zip › jcm-1583915 suppl/Supplementary Table S2.pdf]

Supplementary Table S2. Quality assessment using the Cochrane Risk of Bias Tool for randomized controlled trials.

**First author, year**	**Adequate random sequence generation**	**Allocation concealment**	**Blinding of participants**	**Blinding of personnel**	**Blinding of outcome assessors**	**Incomplete data outcome**	**Selective reporting**	**Other**
Barakat, 2016	+	+	-	+	?	+	+	+
Bhartia, 2019	?	?	-	?	?	+	+	?
de Oliveria, 2012	+	+	-	-	+	+	+	+
da Silva, 2017	+	+	-	-	+	+	+	+
Haakstad, 2016	+	+	+	+	+	+	+	+
Jayashree, 2013	+	?	-	+	+	+	+	+
Kasawara, 2013	+	+	-	?	?	+	+	?
Maharana, 2013	+	?	-	+	+	+	+	+
Perales, 2020	+	?	-	+	+	+	+	+

Price, 2012	+	+	-	?	?	+	+	?
Ruiz, 2013	+	?	-	-	?	+	+	?
Stafne, 2012	+	+	-	-	+	+	+	?
Rakhshani, 2013	?	?	-	+	+	+	+	+
Tomic, 2013	?	?	-	?	?	+	+	?
Wang, 2017	+	+	-	-	?	+	+	?
Yeo, 2000	?	?	-	?	?	+	+	?
