# Supplementary material for: Effects of Supervised Exercise on the Development of Hypertensive Disorders of Pregnancy: A Systematic Review and Meta-Analysis
Source: J Clin Med. 2022 Feb 1;11(3):793. doi: 10.3390/jcm11030793 (PMC8836524; doi:10.3390/jcm11030793)
Supplement: Supplementary file 1 [file jcm-11-00793-s001.zip › jcm-1583915 suppl/Supplementary Table S3.pdf]

Supplementary Table S3. Assessment of the risk of bias for each important outcome within studies.

**Study**	**Risk of bias**	**Interpretation**
Barakat, 2016	Low risk of bias for all key domains.	Plausible bias unlikely to seriously affect the results.
Bhartia, 2019	Unclear risk of bias for one or more domains.	Plausible bias that raises some doubt about the results.
de Oliveria, 2012	Low risk of bias for all key domains.	Plausible bias unlikely to seriously affect the results.
da Silva, 2017	Low risk of bias for all key domains.	Plausible bias unlikely to seriously affect the results.
Haakstad, 2016	Low risk of bias for all key domains.	Plausible bias unlikely to seriously affect the results.
Jayashree, 2013	Low risk of bias for all key domains.	Plausible bias unlikely to seriously affect the results.
Kasawara, 2013	Low risk of bias for all key domains.	Plausible bias unlikely to seriously affect the results.
Maharana, 2013	Low risk of bias for all key domains.	Plausible bias unlikely to seriously affect the results.
Perales, 2020	Low risk of bias for all key domains.	Plausible bias unlikely to seriously affect the results.
Price, 2012	Unclear risk of bias for one or more domains.	Plausible bias that raises some doubt about the results.
Ruiz, 2013	Unclear risk of bias for one or more domains.	Plausible bias that raises some doubt about the results.
Stafne, 2012	Low risk of bias for all key domains.	Plausible bias unlikely to seriously affect the results.
Rakhshani, 2013	Low risk of bias for all key domains.	Plausible bias unlikely to seriously affect the results.
Tomic, 2013	Unclear risk of bias for one or more domains.	Plausible bias that raises some doubt about the results.
Wang, 2017	Unclear risk of bias for one or more domains.	Plausible bias that raises some doubt about the results.
Yeo, 2000	Unclear risk of bias for one or more domains	Plausible bias that raises some doubt about the results.
