# Supplementary material for: Effects of Supervised Exercise on the Development of Hypertensive Disorders of Pregnancy: A Systematic Review and Meta-Analysis
Source: J Clin Med. 2022 Feb 1;11(3):793. doi: 10.3390/jcm11030793 (PMC8836524; doi:10.3390/jcm11030793)
Supplement: Supplementary file 1 [file jcm-11-00793-s001.zip › jcm-1583915 suppl/Supplementary Table S4.pdf]

Supplementary Table S4: Characteristics of the included studies.

Study	Country	Setting	Number of participants	Mean age (years)	Mean BMI (kg/m2)	Ethnicity	Type of HDP	Type of exercise	Weeks' gestation	Supervision frequency	Cumulative Incidence (%) in intervention group	Cumulative Incidence (%) in control group
**Barakat, et al. 2016**	Spain	hospital	765	31.7	23.5	White	GH, PE	aerobic, strength, flexibility	9 to 38-39	3 per week	3 (1,5)	8 (6,11)
**Bhartia et al., 2019**	India	hospital	78	n/a	n/a	Indian	GH	yoga	20 to 32	1 per week	5 (1,7)	10 (4,23)
**de Oliveria et al., 2012**	Brazil	university	171	24.7	23.9	n/a	PE	aerobic	13-20 to 32	3 per week	8 (4,14)	9 (4,19)
**da Silva et al., 2017**	Brazil	university	639	27.1	25.2	Mixed	PE	aerobic, strength, flexibility	16-20 to 32-36	3 per week	2 (1,5)	5 (3,8)
**Haakstad et al., 2016**	Norway	university	61	32.3	22.9	n/a	PIH	aerobic, strength, flexibility	17.6 to 36.5	2 per week	8 (4,14)	8 (2,24)
**Jayashree et al., 2013**	India	hospital	68	27.4	31	Indian	GH, PE, E	yoga	12 to 36	3 per week	10 (3,36)	32 (19,47)
**Kasawara et al., 2013**	Brazil	hospital	116	31.5	33.1	Mixed	PE	aerobic	12-20 to term	1 per week	14 (7,25)	14 (7,25)
**Maharana et al., 2013**	India	hospital	96	25.7	25	Indian	GH	yoga	18-20 to term	1 per week	6 (2,16)	11 (5,23)
**Perales et al., 2020**	Spain	hospital	1348	31.5	23.5	White	GH	aerobic, strength, flexibility	9 to 38-39	3 per week	0 (0,1)	3 (2,4)
**Price et al., 2012**	USA	hospital	62	29.1	27.7	Mixed	GH	aerobic, strength, flexibility	12-14 to 36 or term	4 per week	2 (0,14)	10 (3,25)
**Rakhshani et al., 2013**	India	hospital	68	27.5	25.3	Indian	GH, PE, E	yoga	12 to 28	3 per week	10 (3,26)	45 (30,60)
**Ruiz et al., 2013**	Spain	hospital	962	31.7	23.6	n/a	GH	aerobic, strength, flexibility	9 to 38-39	3 per week	3 (2,5)	6 (4,9)
**Stafne et al., 2012**	Norway	hospital	855	30.5	24.8	White	GH, PE	aerobic, strength, flexibility	20 to 36	1 per week	6 (4,9)	3 (1,5)
**Tomic et al., 2013**	Crotia	multiple	334	29.1	22.9	White	GH, PE	aerobic	6-8 to delivery	3 per week	3 (1,7)	4 (2,8)

**Wang et al., 2017**	China	hospital	300	32.3	26.8	Asian	GH, PE	aerobic	12 to 36-37	3 per week	13 (8,19)	15 (10,21)
**Yeo et al., 2000**	USA	university	16	30	n/a	Mixed	GH	aerobic	18 to 28	3 per week	13 (2,47)	13 (2,47)
