# Supplementary figures and images for: Effects of Supervised Exercise on the Development of Hypertensive Disorders of Pregnancy: A Systematic Review and Meta-Analysis
Source: J Clin Med. 2022 Feb 1;11(3):793. doi: 10.3390/jcm11030793 (PMC8836524; doi:10.3390/jcm11030793)

**Supplementary Figure S2.** Funnel plot for publication bias.

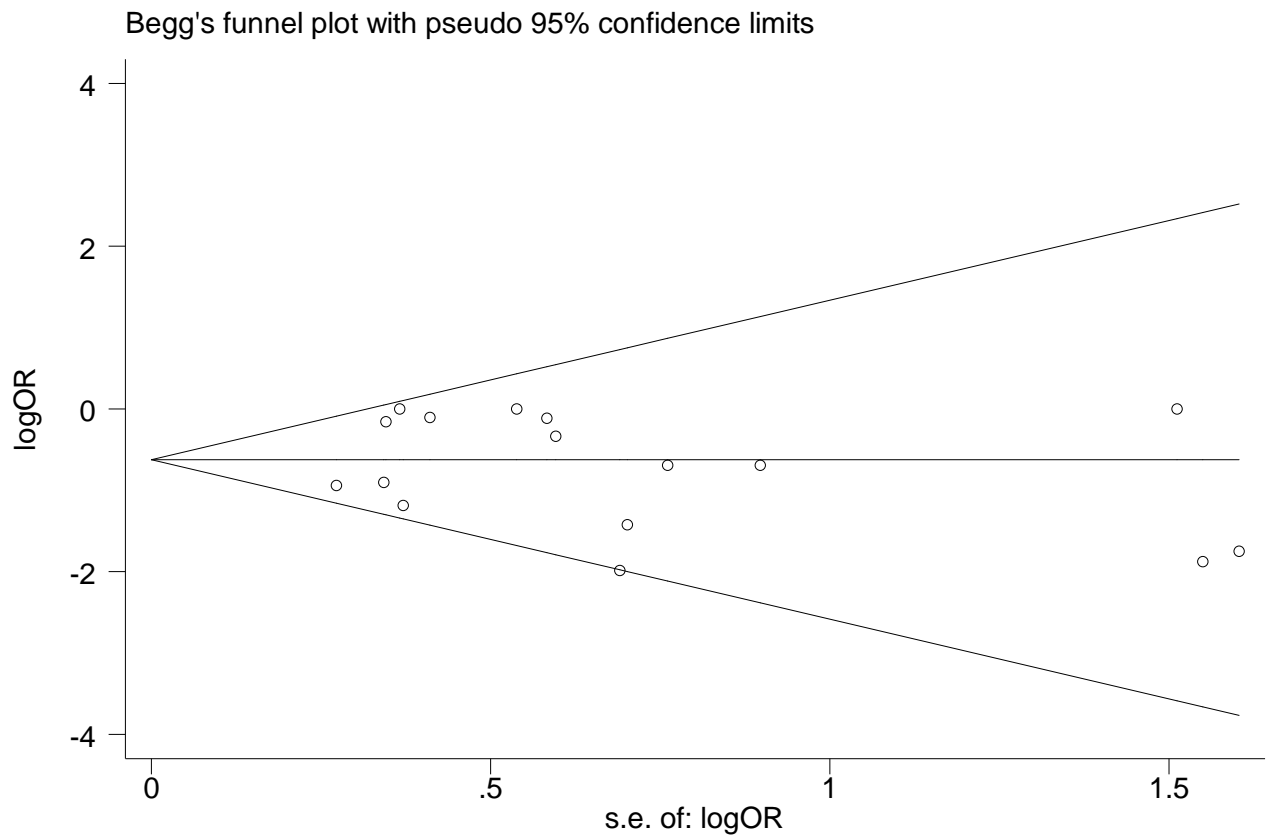

Supplement: Supplementary file 1 [file jcm-11-00793-s001.zip › jcm-1583915 suppl/Supplementary Figure S2.pdf]
